# Supplementary material for: Dental biorhythm is associated with adolescent weight gain
Source: Commun Med (Lond). 2022 Aug 22;2:99. doi: 10.1038/s43856-022-00164-x (PMC9395425; doi:10.1038/s43856-022-00164-x)
Supplement: Supplementary file 1 — Description of Additional Supplementary Files [file 43856_2022_164_MOESM1_ESM.pdf]

Description of Additional Supplementary Files

File Name: Supplementary Data 1

Description: Data for Figure 2a-d

File Name: Supplementary Data 2

Description: Data for Figure 3a-f

File Name: Supplementary Data 3

Description: Data for Figure 4a-f

File Name: Supplementary Data 4

Description: Data for Figure 5a-h
