# Supplementary material for: Dental biorhythm is associated with adolescent weight gain
Source: Commun Med (Lond). 2022 Aug 22;2:99. doi: 10.1038/s43856-022-00164-x (PMC9395425; doi:10.1038/s43856-022-00164-x)
Supplement: Supplementary file 6 — Supplementary Information [file 43856_2022_164_MOESM6_ESM.pdf]

Supplementary Materials

Dental biorhythm is associated with adolescent weight gain

Patrick Mahoney[1], Gina McFarlane[1], Carolina Loch[2], Sophie White[2], Bruce Floyd[3], Erin Dunn[4], Rosie Pitfield[1], Alessia Nava[1], Debbie Guatelli-Steinberg[5]

[1]School of Anthropology and Conservation, University of Kent, Canterbury, UK.
[2]Sir John Walsh Research Institute, Faculty of Dentistry, University of Otago, Dunedin, New Zealand
[3]School of Social Sciences, University of Auckland, New Zealand.
[4]Department of Psychiatry, Harvard Medical School, Boston, MA, USA.
[5]Department of Anthropology, The Ohio State University, Columbus, Ohio, USA.

Supplementary materials include:

1. Supplementary figures

2. Supplementary tables

SUPPLEMENTARY FIGURES

Supplementary Figure 1: Weight gained over 14 months related to height, lower leg length and starting age. (a) Weight gain significantly correlated with height. One outlier removed. (b) Weight gain significantly correlated with lower leg length. (c) Weight gain does not relate to starting age. Pearsons correlation. *p < 0.05, **p < 0.01. Source data are provided as a Source data file.

a

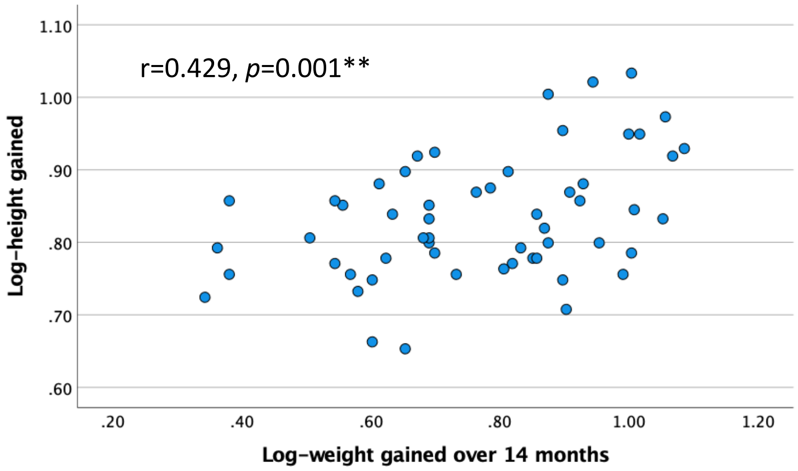

b

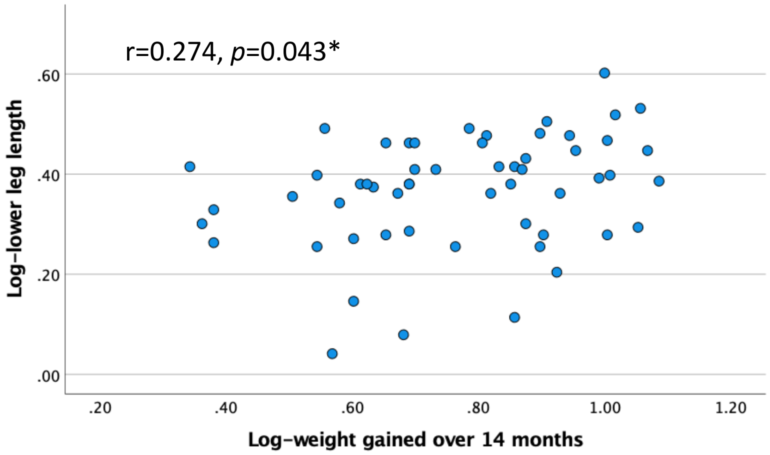

c

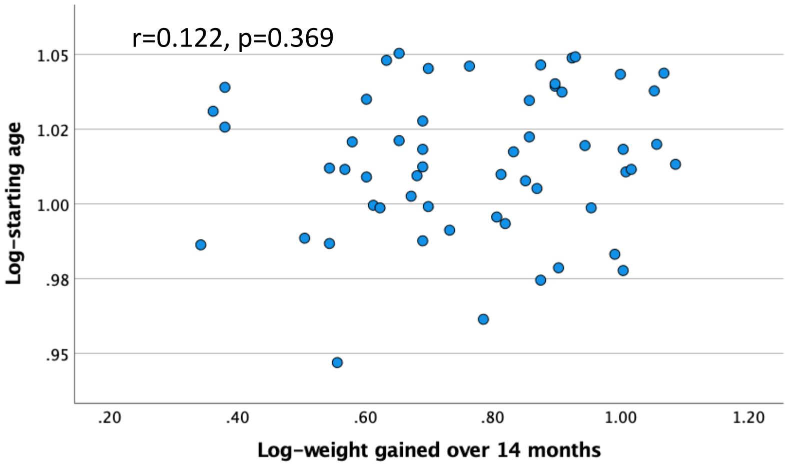

Supplementary Figure 2: Log transformed Weight/BMI gained over 14 months related to maturity scores. (a) Weight gained significantly and positively correlates with maturity scores. (b) BMI gain significantly and positively correlates with maturity scores. One outlier removed. Pearsons correlation. (a-b) *p < 0.05. Source data are provided as a Source data file.

a

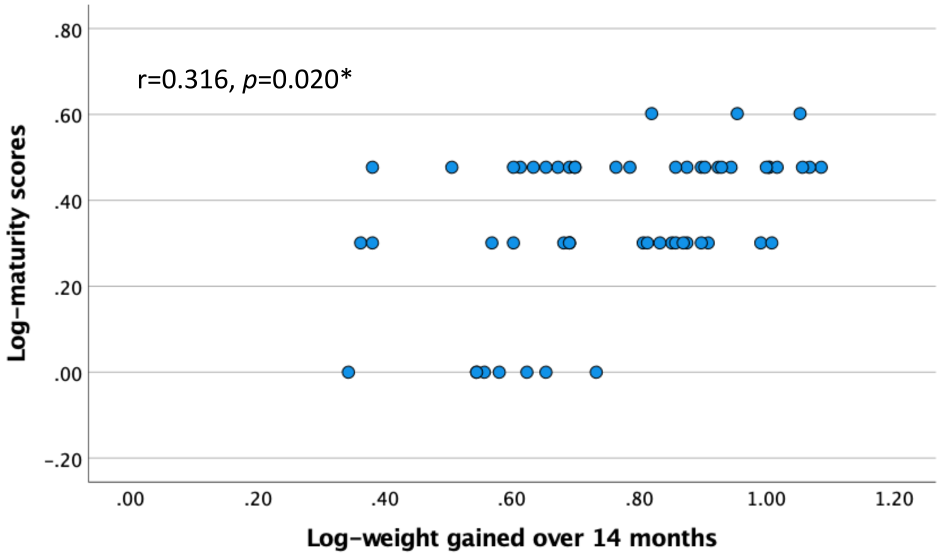

b

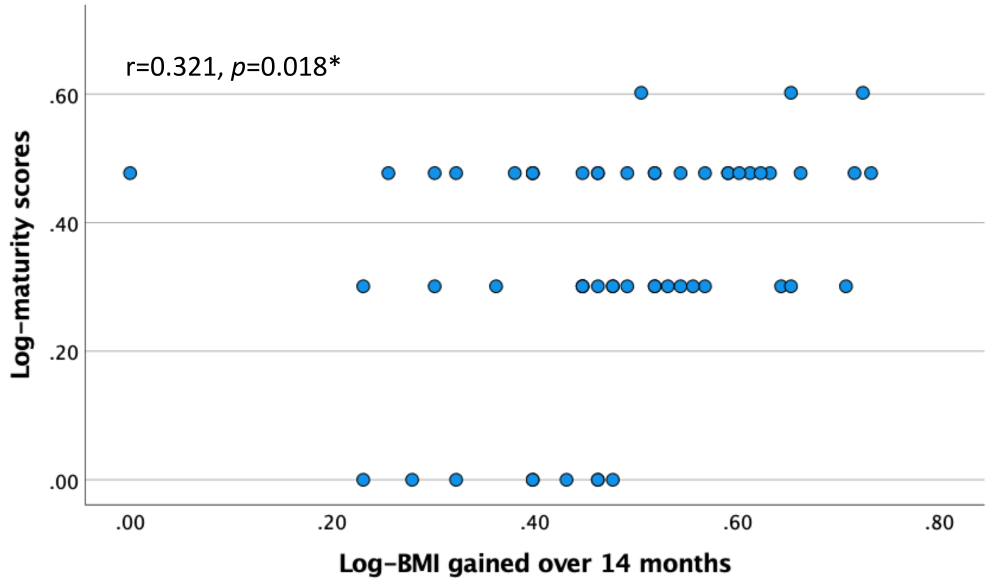

Supplementary Figure 3: Retzius periodicity. (a) Distribution of RP values for female molars. Modal RP for females was eight days. (b) Distribution of RP values for male molars. Modal RP for males was six days. See Table 1 in main text for descriptive statistics.

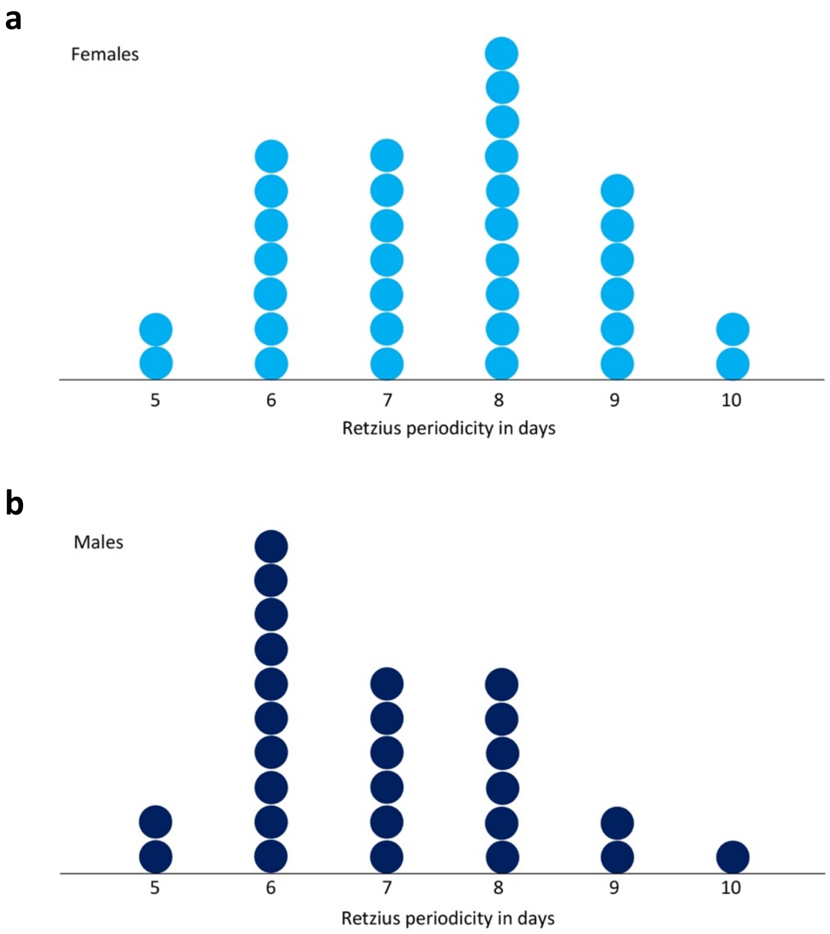

Supplementary Figure 4: Females with maturations scores of 3 related to RP. Scatter plot illustrating the significant relationship between log-transformed weight gained after 14 months for females with maturation scores of 3, and log-RP, using a quadratic regression model.

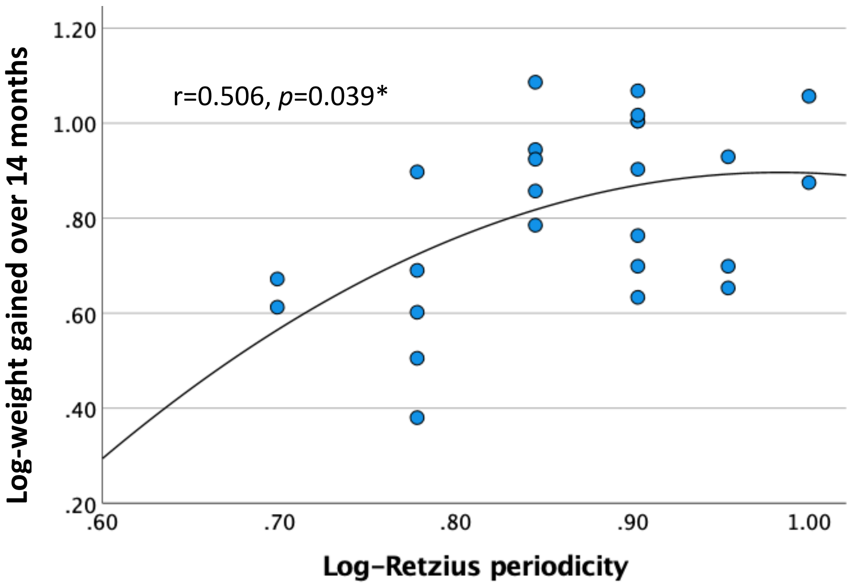

SUPPLEMENTARY TABLES

Supplementary Table 1. Comparing Retzius periodicity between deciduous molars within an individual.

First RP	Second RP	Tooth type first RP	Tooth type second RP
6	6	Lower left dm1	Upper right dm1
10	10	Lower left dm2	Upper right dm2
6	6	Lower right dm1	Lower right dm2
8	8	Lower right dm1	Upper left dm1
6	6	Lower left dm1	Lower left dm2
6	6	Lower left dm1	Lower right dm2
7	7	Upper right dm1	Upper right dm2
7	7	Upper left dm2	Upper right dm2
7	7	Upper left dm2	Upper right dm2
7	7	Lower left dm2	Upper right dm2
6	6	Lower right dm2	Upper right dm2
6	6	Lower right dm1	Lower right dm2
8	8	Lower left dm1	Upper left dm1

Supplementary Table 2. Partial correlations. The table shows that when controlling for the influence of starting age on the relationship between RP and weight gained over 14 months, the relationship is significant, but the controlling effect is minimal. This is supported by the very weak correlations between RP and starting age, and when weight gain is compared to starting age.

	**Variable**		**Log-RP**	**Log-weight gained**	**Log-starting age**
	Log-RP	Correlation	1.000	0.403	0.034
		Significance		0.002*	0.799
	Log-weight gained	Correlation	0.403	1.000	-0.060
		Significance	0.002*		0.659
	Log-starting age	Correlation	0.034	-0.060	1.000
		Significance	0.799	0.659	
**Controlling variable**					
Log-starting age	Log-RP	Correlation	1.000	0.406	
		Significance		0.002*	
	Log-weight	Correlation	0.406	1.000	
		Significance	0.002		

Supplementary Table 3. Average weight gained in kg related to seasons and lockdown[1]

**Biorhythm**	**Spring**<br>Sept - Nov	**Summer**<br>Dec - Feb	**Lockdown[2]**<br>End Feb - June	**Winter**<br>June - Aug	**Spring**<br>Sep - Nov
6	0.61	0.99	1.00	0.60	0.57
7[1]	1.21	1.10	3.50	1.30	1.01
8	0.90	0.70	3.03	1.49	1.00

1=Using paired values only. 2=Last measurement in February: a 4-month period, not 3 months like the seasons. 3=Excludes one outlier more than 60kg.
