# Supplementary material for: Dental biorhythm is associated with adolescent weight gain
Source: Commun Med (Lond). 2022 Aug 22;2:99. doi: 10.1038/s43856-022-00164-x (PMC9395425; doi:10.1038/s43856-022-00164-x)
Supplement: Supplementary file 8 — Reporting Summary [file 43856_2022_164_MOESM8_ESM.pdf]

nature research

Corresponding author(s): Patrick Mahoney
Last updated by author(s): 13/6/22

Reporting Summary

Nature Research wishes to improve the reproducibility of the work that we publish. This form provides structure for consistency and transparency in reporting. For further information on Nature Research policies, see our Editorial Policies and the Editorial Policy Checklist.

Statistics

For all statistical analyses, confirm that the following items are present in the figure legend, table legend, main text, or Methods section.

n/a	Confirmed	
☐	☒	The exact sample size (n) for each experimental group/condition, given as a discrete number and unit of measurement
☐	☒	A statement on whether measurements were taken from distinct samples or whether the same sample was measured repeatedly
☐	☒	The statistical test(s) used AND whether they are one- or two-sided<br>*Only common tests should be described solely by name; describe more complex techniques in the Methods section.*
☐	☒	A description of all covariates tested
☐	☒	A description of any assumptions or corrections, such as tests of normality and adjustment for multiple comparisons
☐	☒	A full description of the statistical parameters including central tendency (e.g. means) or other basic estimates (e.g. regression coefficient) AND variation (e.g. standard deviation) or associated estimates of uncertainty (e.g. confidence intervals)
☐	☒	For null hypothesis testing, the test statistic (e.g. F, t, r) with confidence intervals, effect sizes, degrees of freedom and P value noted<br>*Give P values as exact values whenever suitable.*
☒	☐	For Bayesian analysis, information on the choice of priors and Markov chain Monte Carlo settings
☒	☐	For hierarchical and complex designs, identification of the appropriate level for tests and full reporting of outcomes
☒	☐	Estimates of effect sizes (e.g. Cohen's d, Pearson's r), indicating how they were calculated

Our web collection on statistics for biologists contains articles on many of the points above.

Software and code

Policy information about availability of computer code

Data collection	N/A
Data analysis	IBM Corp. Released 2019. IBM SPSS Statistics for Windows, Version 26.0. Armonk, NY: IBM Corp

For manuscripts utilizing custom algorithms or software that are central to the research but not yet described in published literature, software must be made available to editors and reviewers. We strongly encourage code deposition in a community repository (e.g. GitHub). See the Nature Research guidelines for submitting code & software for further information.

Data

Policy information about availability of data

All manuscripts must include a data availability statement. This statement should provide the following information, where applicable:
- Accession codes, unique identifiers, or web links for publicly available datasets
- A list of figures that have associated raw data
- A description of any restrictions on data availability

Data supporting Figures 2, 3, 4 and 5 are present in four Supplementary Data Files. All data supporting this study described in this manuscript are available at University of Kent data repository. URL to the site provided in manuscript.

Field-specific reporting

Please select the one below that is the best fit for your research. If you are not sure, read the appropriate sections before making your selection.

☒ Life sciences ☐ Behavioural & social sciences ☐ Ecological, evolutionary & environmental sciences

For a reference copy of the document with all sections, see nature.com/documents/nr-reporting-summary-flat.pdf

Life sciences study design

All studies must disclose on these points even when the disclosure is negative.

Sample size	Sample size was n=61 (34 females; 27 males). The 61 participants (n=34 females and n=27 males) were selected from a larger cohort that were part of the Biorhythm of Childhood Growth project. Selection was based upon histology criteria (below).
Data exclusions	Each participant was selected for inclusion into the study if we were able to produce two matching Retzius periodicity values for their primary molars.
Replication	A Retzius periodicity value was calculated for each individual. If we were able to replicate the first value for the same individual, they were included in the study. All height, weight and lower leg length measurements were taken three times, from each individual, each month. Values reported in the raw data set are means of the three values.
Randomization	Retzius periodicity values were related to gender, weight gain, and a mass greater or less than the 95th percentile. The relative effect of the covariates height, lower leg length, and maturity on the predictor variable (Retzius periodicity) was examined through standardized beta coefficients. The potential effects of a Covid-19 lockdown, and season of the year, were also assessed.
Blinding	The raw data sets for Retzius periodicity, and growth measurements (height, weight, and lower leg length), were recoded independently and blind of each other, in the United Kingdom and New Zealand, respectively.

Reporting for specific materials, systems and methods

We require information from authors about some types of materials, experimental systems and methods used in many studies. Here, indicate whether each material, system or method listed is relevant to your study. If you are not sure if a list item applies to your research, read the appropriate section before selecting a response.

Materials & experimental systems

n/a	Involved in the study	
☒	☐	Antibodies
☒	☐	Eukaryotic cell lines
☒	☐	Palaeontology and archaeology
☒	☐	Animals and other organisms
☐	☒	Human research participants
☒	☐	Clinical data
☒	☐	Dual use research of concern

Methods

n/a	Involved in the study	
☒	☐	ChIP-seq
☒	☐	Flow cytometry
☒	☐	MRI-based neuroimaging

Human research participants

Policy information about studies involving human research participants

Population characteristics	Participants (34 females; 27 males) were from the same school-year (average starting age = 10.33yrs) and are enrolled in the Biorhythm of Childhood Growth (BCG) project. The BCG is an ongoing prospective cohort study that investigates childhood development in middle-income children from Dunedin, New Zealand.
Recruitment	Participation in this study was based upon histology criteria. Each participant was selected for inclusion into the study if we were able to produce two matching Retzius periodicity values for their primary molars.
Ethics oversight	University of Otago, Human Ethics Committee (approval number H19/030). Research consultation with Māori was obtained from the Ngāi Tahu Research Consultation Committee.

Note that full information on the approval of the study protocol must also be provided in the manuscript.
